# Using technology to support clinical care and research in rheumatoid arthritis

**DOI:** 10.1097/BOR.0000000000000485

**Published:** 2018-02-01

**Authors:** William G. Dixon, Kaleb Michaud

**Affiliations:** a Arthritis Research UK Centre for Epidemiology, Division of Musculoskeletal and Dermatological Sciences, The University of Manchester; b Division of Rheumatology and Immunology, Department of Medicine, University of Nebraska Medical Center, Omaha, Nebraska and The National Databank for Rheumatic Diseases, Wichita, Kansas, USA

**Keywords:** health information technology, mobile health, outcome measures, rheumatoid arthritis

## Abstract

**Purpose of review:**

As digital technology becomes more ubiquitous, understanding the current state-of-the-art in digital information use for clinical care and research for patients with rheumatoid arthritis (RA) is timely and relevant.

**Recent findings:**

The opportunities for recording and utilizing high-quality data from rheumatologists are reviewed, as well as opportunities from collecting, integrating and analysing patient-generated data to deliver a step-change in the support and management of RA.

**Summary:**

Once greater adoption, standardization and implementation of relevant RA measures are in place within electronic health records (EHRs), patient care will improve and the ability to learn from aggregate experiences increases dramatically. Incorporating passive and patient-reported outcomes into self-management apps and integrating such data into the patient's health record will provide more responsive and better treatment results.

## INTRODUCTION

Since the introduction of the World Wide Web 25 years ago, we are living in the ‘Information Age’ or ‘Digital Age’, a period of human history characterized by an economy based on information computerization [1]. Advances in technology have transformed health care, alongside other industries, through innovations such as electronic health records (EHRs), digital imaging, wireless sensors and access to online information. These changes touch the majority of our lives: for example, over 80% of Internet users seek health information online [2]. Increasing numbers of people own mobile devices from which they access the Internet. In the United States, over 95% of adults own a mobile phone [3^▪▪^] and over seven in 10 UK adults owns a smartphone, with older people more recently embracing smart and social technology [4]. Rheumatology and other clinical specialities need to adapt to this changing environment, embracing opportunities that emerge from better digital data and information. As rheumatoid arthritis (RA) remains a cornerstone of rheumatology practice, we review these advances in technology and their opportunities with an RA focus.

RA is a long-term condition in which symptoms including joint pain and difficulty with daily tasks vary over time and can progress to joint deformity. Treatment paradigms have changed in response to evidence from clinical trials and observational data. With the advent of biologic therapies and treat-to-target approaches seeking remission, prospects for patients are much better compared with previous decades. Nonetheless, we continue to strive to improve care and better understand treatment choices. Data are a powerful tool in advancing our knowledge, and technology has the potential to transform what data we can collect about RA and how it is presented to advance care. With careful consideration about what data clinicians, patients and others collect and how it is captured, its use can expand beyond clinical care to research, audit, quality improvement and more. The new era of digital epidemiology has a huge opportunity to transform our understanding of disease and treatment through more granular data and advanced analytics, resulting in improved information for medical decision-making [5].

The current article will describe opportunities for using technology to support self-management, clinical care and research in RA, as well as noting some important barriers. The two main topics that will be discussed include collecting and utilizing high-quality data from clinicians, and opportunities for collecting, integrating and analysing patient-generated data to deliver a step-change in the support and management of RA. 

**Box 1:**
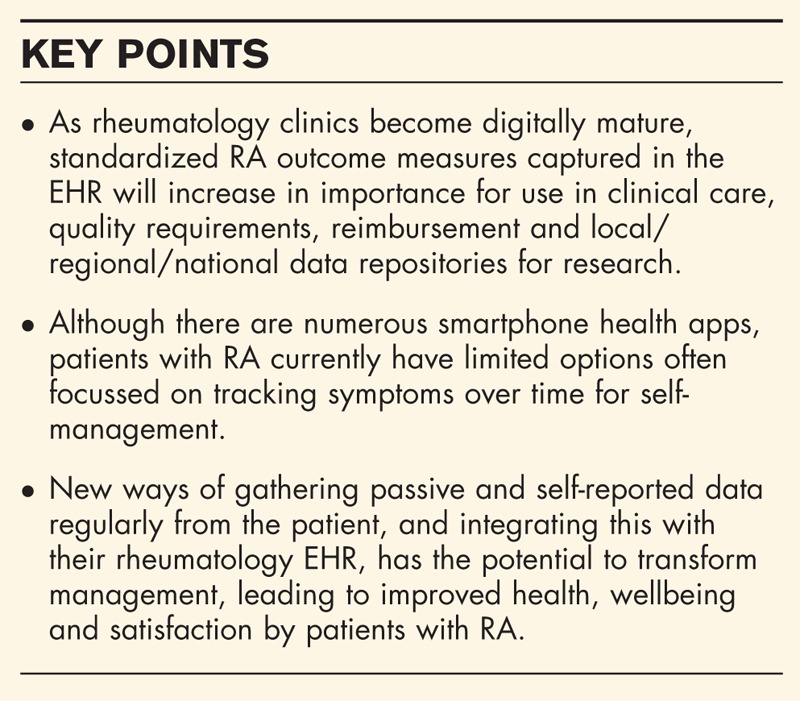
no caption available

## CLINICIAN-DERIVED DATA

The uptake of EHRs is increasing across the healthcare industry. In the United Kingdom, nearly all primary care practices are digitized, whereas a recent review suggested all hospitals should reach ‘digital maturity’ by 2023 [6]. The increasing use of EHRs is an important opportunity for rheumatologists to record and access better data about their patients to support improved clinical care, national audit, quality improvement programmes, research and more. As the data required for each of these purposes overlap, it is conceivable that rheumatologists might collect data once and use it to support all of these areas. The many benefits will flow more easily if data are collected in a structured and standardized way. Uptake will be enhanced if systems are useable and indeed useful in supporting rheumatologists to care for their patients in the best possible way. Careful thought thus needs to go into the design and implementation of such systems, but there are already examples of best practice from which we can learn in RA, such as the DANBIO register (Table 1).

Although DANBIO gives one illustration of what is possible in the use of structured data within EHRs for clinical care and research, data are typically collected in different ways in different systems. This can make it challenging to pool data resources when conducting large population research, or to support national audit. A review of 25 European RA cohorts found heterogeneity both in *what* was collected and *how* it was collected [11]. For example, although all cohorts collected information on disease severity, the instrument to measure disease severity varied with 80% including disease activity score-28 (DAS28) and 40% Clinical Disease Activity Index. There was greater variability in the collection of other data such as physical function, fatigue, comorbidities and radiological damage [11]. In the United States, greater disparities exist with many items not being collected systematically [12] despite there being established quality care indicators [13].

A European League Against Rheumatism (EULAR) taskforce has recently agreed upon a core dataset for RA that was designed, importantly, to support both clinical care *and* research. Data items were selected which would be useful and feasible to collect in real-time clinical practice within EHRs, and which would also support high-quality observational research [14]. Similarly, the American College of Rheumatology (ACR) has task forces leading recommendations on RA activity measures and physical function assessment measures to help reduce the heterogeneity in EHR vendors’ systems [15].

Maximizing the use of RA EHR data, once collected, is a challenge being addressed in a number of settings. The ACR recently launched its Rheumatology Informatics System for Effectiveness (RISE) registry that passively updates its data repository with connected rheumatology EHRs. Participating clinics can view their performance on a number of quality improvement measures in comparison with others while simultaneously complying with US reporting requirements [13,16]. Research is planned within RISE, but there are important limitations when trying to analyse hundreds of clinics with different EHRs and data collected. Data quality is variable: the RISE group has noted that measurement of RA activity was more likely if a clinic had been participating for longer [17]. RISE is also developing methods to extract value from unstructured data whilst awaiting improvements in structured data collection [18]. Recognizing the limitations of what research is possible even if you have data from whole countries, a pan-Nordic rheumatology register is being piloted to link individual-patient data across national borders without physical data transfer [19].

Clinical records have supported patient management and research for decades. Their increasing digitization provides important opportunities to deliver a step-change in how we manage patients with RA effectively and safety. The digital era also has the potential to shift from the paradigm of information coming solely from clinicians, to now supplement this clinician-generated data with information that comes directly from patients.

## PATIENT-GENERATED DATA

The uptake of consumer technology including smartphones, smartwatches and wearables into patients’ lives generates a range of digital opportunities for clinical care and research in RA. These include the collection of patient-generated data to support both self-management through symptom tracking and to inform clinical decisions if integrated successfully into clinical workflows; the use of sensors and wearables to measure and track important outcomes such as physical activity; and digital interventions such as behaviour change nudges.

## TECHNOLOGY TO SUPPORT SELF-MONITORING AND SELF-MANAGEMENT

Patients spend over 99% of their time outside of the clinical environment and therefore often need to self-manage their RA. Specific self-management methods include rest, pacing and exercise; technical aids that address occupational and daily productivity; and pain management through self-medication. A variety of linked comorbidities also require active self-management by the patient. The use of consumer technology to track symptoms has the power to improve self-management in RA.

There are currently more than 165 000 health apps available in Apple's App Store [20], many of them designed to allow patients to monitor their disease through journaling or logging behaviours and symptoms [21]. Data entry is typically self-reported information; although inclusion of other data sources such as camera images, within-device physical activity tracking and wireless linkage to other devices such as blood pressure cuffs is increasingly common. Patterns through time are often presented back graphically to the user [22]. Short-term benefits of symptom tracking across disease areas include understanding disease and symptoms, acceptance, identifying triggers and reducing anxiety [23^▪▪^]. The evidence base for benefits in hard clinical outcomes such as a reduction in disease severity across disease areas, however, is less convincing for self-monitoring alone. Findings in chronic obstructive pulmonary disease and heart failure are debated, and evidence is equivocal in hypertension and diabetes [24–27].

## APPS FOR PATIENTS WITH RHEUMATOID ARTHRITIS

A recent search identified 19 apps dedicated to RA, although the number continues to expand [28^▪^]. RA apps broadly divided into those that provided calculators for rheumatologists, for example to calculate a DAS28 score, and apps that allowed patients to track symptoms. The authors sought to examine to what extent patient data collection used validated tools and scores, concluding that they ‘do not uniformly collect data using validated instruments or composite disease activity measures’ [28^▪^]. It should be noted, however, that such instruments were developed for a different primary purpose (i.e. not for regular reporting of patient-generated data), and so the use of new measures might be expected. This is particularly true if retaining participant engagement is a goal.

There is limited evidence to date about the benefits of symptom tracking in RA. In our own experience (currently unpublished), we have observed patients’ self-management benefit from tracking symptoms through increased insight into changes in their disease through time, identifying triggers, informing pacing, as well as improving communication about disease with family and friends. The use of digital interventions within a smartphone app also holds significant promise. Interventions might include providing accessible patient and carer information, for example about RA or immunosuppressive medication; behaviour change support such as physical activity guidance or medication adherence; or support for improving emotional wellbeing such as online cognitive behavioural therapy for depression or sleep disturbance [29], or peer-to-peer support through online communities [30^▪^].

## INTEGRATING PATIENT-GENERATED DATA INTO CLINICAL PRACTICE

### Assessment at clinic visits

Patient-reported outcomes are well established as being important in RA clinical care and research: the DAS28 score includes a patient global assessment [31] and the ACR/EULAR core outcome set for RA clinical trials includes a measure of fatigue [32]. The uptake of self-reported questionnaires in clinical practice, however, has been somewhat limited, in part due to their perceived usefulness by some clinicians as well as practicalities of administration and scoring [33]. Technology has the potential to simplify the administrative burden and to integrate patient-generated data into clinical workflows. In rheumatology clinics across Denmark, patients all report symptoms on touch screens prior to joining the consultation with around 90% completeness [7] (Table 1). In Sweden, patients are able to report their symptoms prior to their consultation in the waiting area or from home (see https://www.youtube.com/watch?v=Kmqzy1hqcOw). Although some clinicians may not trust patient-reported data over their own assessment, studies have demonstrated patient reports to be well correlated with clinician assessments [34,35^▪^].

### Daily assessment and remote monitoring

Treatment decisions are made in response to patients’ descriptions of their symptoms when they see a health professional, which may be every 3–6 months. An accurate picture, however, can be obscured by patients’ willingness to discuss symptoms, eloquence, recall, stoicism, the influence of recent disease severity and more [36,37]. This means treatment decisions are made using information that is imperfect, in turn suggesting decision-making may be suboptimal. Remote monitoring using consumer technology could be transformative in providing a clearer picture of disease through time if it could be integrated into clinical practice. In a recent review about opportunities in RA, it was argued that remote monitoring would potentially improve disease control [38]. A ‘treat to target’ paradigm with a target of remission is accepted in RA [39], yet it is often not feasible for clinicians to review patients monthly as advocated in guidelines [40]. At present, though, it is rare that patient-generated data are successfully integrated into clinical systems: a consequence of multiple challenges including patient and provider concerns, technical and workflow issues and privacy and security requirements [41].

We anticipate, however, that all such challenges are surmountable in the coming years. Rheumatologists can expect to view a clear picture of how disease severity has changed since the patients’ last visit within their EHR before too long. Our own experience in a pilot study of remote monitoring in RA is that such integrated remote monitoring data are both feasible and useful, holding significant promise for clinicians and patients [42^▪^] (see also http://www.cfe.manchester.ac.uk/research/projects/remora/). Additional future opportunities include using remote monitoring data for rationalizing appointments [43] and triggering remote consultations, making service delivery more efficient. We are involved with a pilot study, testing if a smartphone app can help detect and provide prompt follow-up of flares between clinical visits (Wang *et al.*, under review JMIR Research Protocols).

## PATIENT-GENERATED DATA FOR RESEARCH

Daily data collected as part of remote monitoring has the potential to address important research questions that have been impossible to answer to date. They will allow exploration of day-to-day patterns of disease fluctuation. The effectiveness of treatment can be studied by uniquely charting the rapidity and trajectory of response rather than being limited to assessing change between two distant time points. This advance would allow doctors to preferentially prescribe treatments that have a quicker onset of action. Furthermore, daily symptoms collected in the run-up to a disease flare would allow identification of a preflare period, supporting the development and assessment of a (potentially digital) intervention to prevent, or improve the management of, the approaching flare.

## PASSIVE MONITORING

Regular remote monitoring using patient-generated data has much appeal, and yet it is hard to conceive that high proportions of patients will remain engaged in remote monitoring for many years. We have evidence that motivated patients will track symptoms on a daily basis for 6 months or more [44], but it is likely that reporting fatigue will set in at some point. There are, nonetheless, important opportunities for passive monitoring of disease severity using technology that could support long-term remote monitoring. This might include the use of physical activity monitoring, given the known relationship between increasing disease severity and reduction in movement and the inclusion of accelerometers, gyroscopes and Global Positioning Systems in smartphones and other wearable devices along with geofencing tools to detect when a patient visits the hospital [45]. Just by carrying a phone or wearing a sensor, it may be possible to infer information about RA disease severity. Passive monitoring using patterns of physical activity has been explored in neurological conditions [46] and has face validity for RA and musculoskeletal disease. Other emerging methods of monitoring disease passively include examining the ‘digital exhaust fumes’ of our daily lives, in which worsening disease severity may correlate with online search histories or patterns of smartphone use [47,48]. It remains uncertain how well such measures can capture disease severity, although pilot studies show evidence that some passive data collection including mobility, phone call and texting behaviour are associated with self-reported RA disease activity [49]. Furthermore, if they are to be clinically meaningful, we need to be able to convert these data into clinical insight and present in a way that is useful and acceptable to the clinical community [50].

## CONCLUSION

Taken together, the benefits to self-management, clinical care and research from technology have significant opportunities for advancing health and well being at an individual and population level. The path to successful adoption and use, however, has significant challenges including influencing EHR providers to design systems to support disease-specific needs, standardizing data items across geographies with trusted extraction and reuse of health data beyond direct care, up-front investment for longer term gain, maintaining motivation for sustained engagement of data collection, equitable access to digital services and digital literacy, and ensuring interoperability and integration across multiple platforms. Nonetheless, the potential benefits are vast. We are starting to glimpse real transformations in clinical care and research. This is a future worth striving for.

## Acknowledgements

None.

### Financial support and sponsorship

W.G.D.'s work is supported by the Arthritis Research UK Centre for Epidemiology (20380). K.M. is currently receiving grant funding from the Rheumatology Research Foundation and Pfizer.

### Conflicts of interest

There are no conflicts of interest.

## REFERENCES AND RECOMMENDED READING

Papers of particular interest, published within the annual period of review, have been highlighted as:

▪ of special interest
▪▪ of outstanding interest

## Figures and Tables

**Table 1:** Structured rheumatoid arthritis data collection in DANBIO

The Danish biologics registry DANBIO is an EHR system that collects structured data on patients with RA at least once per year, including RA severity. Patients reporting their symptoms via touch screens in the clinic at each visit supplement clinician-reported data. Digital data are summarized graphically and used as a tool for shared informed decision-making between clinicians and patients, for example demonstrating how changes in medication use have correlated with disease severity through time. DANBIO also acts as a quality registry, an audit and feedback tool, and provides secondary use of data for research while fulfilling its primary purpose of supporting clinical care [7]. Examples of research outcomes include the comparative effectiveness of biologic therapies [8], long-term biologic safety [9] and evidence to support automated nudging of treatment intensification [10^▪^]

EHR, electronic health record; RA, rheumatoid arthritis.
